# The Biodiversity of Edible Flowers: Discovering New Tastes and New Health Benefits

**DOI:** 10.3389/fpls.2020.569499

**Published:** 2021-02-22

**Authors:** Stefano Benvenuti, Marco Mazzoncini

**Affiliations:** Department of Agricultural, Food and Agro-Environmental Sciences, University of Pisa, Pisa, Italy

**Keywords:** ethnobotany, food safety, organoleptic perception, nutraceuticals, new foods

## Abstract

Floriculture and horticulture have always been two parallel and very distinct agronomic realities. Floriculture is concerned with meeting the ornamental needs of our urban ecosystems, while horticulture is based on meeting food requirements. These two activities have now converged toward a food chain where flowers are conceived of as a sort of “new vegetable” and one of the most promising novelties to satisfy the growing need for food innovation both in terms of an organoleptic and nutraceutical profile. This novelty has rapidly evolved, especially following the growing scientific evidence of the human health benefits of flowers used as food. The typically high pigment concentration of the corollas (especially flavonoids and carotenoids), which have evolved to chromatically attract pollinators, indicates a marked nutraceutical activity especially in terms of antioxidant power. In this review, we first attempted to explore which species are most promising and which should be avoided due to real or suspected toxicity problems. The nutraceutical virtues were therefore highlighted trying to focus attention on those “functional phytochemicals” capable of counteracting some specific human pathologies. Furthermore, the organoleptic profile of edible flowers was investigated since this is one of the least known aspects. The cropping systems suitable for their cultivation were therefore hypothesized and finally the criticalities of edible flowers were addressed in terms of shelf life and marketing opportunities.

## Introduction

The use of flowers as food is not, however, a new discovery, but a rediscovery of ancient ethnobotanical traditions. In fact, the Greeks and Romans used flowers both to give a surprising ornamental impact to various dishes (for example, rose petals in ancient Rome Melillo, 1994) and to enhance the organoleptic synergy between the taste of traditional foods (vegetables, meat, fish, etc.) and that of flower aromas. However, the gastronomic use of flowers has been limited to and dependent on the relatively narrow, seasonal timeframe in which these flowers proliferate almost exclusively in natural ecosystems. The use of mallow (*Malva sylvestris*), borage (*Borago officinalis*), and acacia (*Robinia pseudoacacia*) flowers are the best known examples of an appreciated but “season-dependent” food potential of flowers. Today this problem of seasonality has been gradually attenuated by an “agronomic conversion” of flower species that were traditionally cropped exclusively as ornamentals toward their cultivation for food purposes. Since this innovative agronomic chain intended for human consumption is recent, it is often not clear which species are actually edible and which are potentially toxic or even poisonous. The benefits, contraindications and potential toxicity of the biodiversity of wild flowers need to be clarified and so that there can be a focus on those suitable for food.

The growing interest in edible flowers is motivated not only by decorative and nutraceutical objectives, but also by the desire for new flavors and new opportunities for gastronomic innovation. The considerable nutraceutical activity in terms of antioxidant power of flowers (Falla et al., 2020; Mikołajczak et al., 2020) derives from their richness in generic phenolic compounds. flavonoids, consisting of flavonols, flavones, and anthocyanins show a strong biological activity. These chemicals play a crucial role in mitigating the oxidative stress induced by various pathologies. flowers are particularly rich in these phytochemicals. Almost all ornamental flowers have evolved chromatically showy corollas as a strategy to attract pollinators [mainly bees, solitary bees, bumblebees, hoverfly (Diptera Syrphidae), bee fly (Diptera Bombyliidae), and butterfly], since they are responsible for the gene flow within each species. The high pigmentation of the flower corollas derives from a co-evolution mechanism with a mutual reward: (i) advertising for pollinators by increasing the degree of seed set and (ii) evolution of the visual system of pollinators thus facilitating the food detection (pollen and / or nectar). This also happens in intensely colored fruits (blackberries, blueberries, raspberries, etc.) in which the pigmentation derives from a flora-fauna co-evolution that facilitates fruit recognition and attractiveness thus leading to frugivorous dispersal. The heterogeneity of the botanical structures of several species of flowers is closely connected with their respective chemical diversity.

The aim of this review was to analyze the “state of the art” of edible flowers in terms of a botanical, phytochemical, nutraceutical, organoleptic, ecological and agronomic profile, as well as to verify their future perspectives in relation to consumer tastes and marketing.

## Flowers as Nutraceutical Food

The analysis of nutraceutical food has increasingly shown that in flower tissues there are a wide range of phytochemicals with positive effects on human health (Table 1). The most well-known parameter of edible flowers is the marked antioxidant activity which is characteristic of almost all the species studied (Kaisoon et al., 2011; González-Barrio et al., 2018; Kalemba-Drożdż and Cierniak, 2019). A high antioxidant activity has been found not only in the floral tissues before their ingestion but also after the digestive processes, highlighting the prolonged bioactive effect of the various phytochemicals (Chen et al., 2015). This antioxidant activity involves the slowing down of cellular aging (Chen et al., 2020), and thus prevent and/or inhibit many pathologies (Lu et al., 2016).

**TABLE 1:** Biodiversity of flower species studied as food.

Scientific name	Botanic family	Traditional use	References
*Agastache foeniculum*	Lamiaceae	Ornamental	Mlcek and Rop, 2011
*Ageratum houstonianum*	Asteraceae	Ornamental	Benvenuti et al., 2016
*Allium schoenoprasum*	Liliaceae	Wild	Kucekova et al., 2013
*Albizia julibrissin*	Fabaceae	Ornamental	Zeng et al., 2014
*Alcea rosea*	Malvaceae	Ornamental	Kalemba-Drożdż and Cierniak, 2019
*Alpinia galanga*	Zinziberaceae	Horticultural	Rachkeeree et al., 2018
*Amomum maximum*	Zinziberaceae	Ornamental	Rachkeeree et al., 2018
*Antigonon leptopus*	Polygonaceae	Ornamental	Kaisoon et al., 2011
*Antirrhinum majus*	Scrophulariaceae	Wild and Ornamental	Benvenuti et al., 2016
*Bauhinia variegata*	Fabaceae	Ornamental	Villavicencio et al., 2018
*Begonia semperflorens*	Begoniaceae	Ornamental	Benvenuti et al., 2016
*Bellis perennis*	Asteraceae	Wild	Kucekova et al., 2013
*Borago officinalis*	Boraginaceae	Wild	Benvenuti et al., 2016
*Bougainvillea hybrid*	Nyctaginaceae	Ornamental	Kaisoon et al., 2011
*Brassica oleracea*	Brassicaceae	Food as Seed Oil	Fernandes et al., 2017
*Calendula officinalis*	Asteraceae	Ornamental and Medicinal	Benvenuti et al., 2016
*Camellia japonica*	Theaceae	Ornamental	Li et al., 2014
*Campanula rapunculus*	Campanulaceae	Wild	Ranfa and Bodesmo, 2017
*Cassia siamea*	Fabaceae	Ornamental	Kaisoon et al., 2011
*Centaurea cyanus*	Asteraceae	Wild and Ornamental	Rop et al., 2012
*Chrysanthemum* spp.	Asteraceae	Ornamental	Ukiya et al., 2001
*Cichorium intybus*	Asteraceae	Wild and Horticultural	Kucekova et al., 2013
*Citrus aurantium*	Rutaceae	Food as Fruit	Kalemba-Drożdż and Cierniak, 2019
*Clitoria ternatea*	Fabaceae	Ornamental	Kaisoon et al., 2011
*Cosmos sulphureus*	Asteraceae	Ornamental	Kaisoon et al., 2012
*Cucurbita pepo*	Cucurbitaceae	Horticultural	Aquino-Bolaños et al., 2013
*Curcuma plicata*	Zingiberaceae	Ornamental	Rachkeeree et al., 2018
*Curcuma sessilis*	Zingiberaceae	Ornamental	Wongwattanasathien et al., 2010
*Dahlia mignon*	Asteraceae	Ornamental	Pires et al., 2018
*Dianthus* x *barbatus*	Caryophyllaceae	Ornamental	Benvenuti et al., 2016
*Echinacea* spp.	Asteraceae	Ornamental and Medicinal	Chen and Wei, 2017
*Etlingera elatior*	Zingiberaceae	Ornamental	Rachkeeree et al., 2018
*Fernaldia pandurata*	Apocynaceae	Ornamental	Morton et al., 1990
*Fuchsia hybrida*	Onagraceae	Ornamental	Benvenuti et al., 2016
*Gerbera jamesonii*	Asteraceae	Ornamental	Li et al., 2014
*Hedychium forrestii*	Zingiberaceae	Ornamental	Rachkeeree et al., 2018
*Hedysarum coronarium*	Fabaceae	Forage	Loizzo et al., 2016
*Hemerocallis* spp.	Asphodeloideae	Ornamental	Mlcek and Rop, 2011
*Hibiscus rosa-sinensis*	Malvaceae	Ornamental	Li et al., 2014
*Hibiscus sabdariffa*	Malvaceae	Food and Fibre	Kalemba-Drożdż and Cierniak, 2019
*Impatiens walleriana*	Balsaminaceae	Ornamental	Rop et al., 2012
*Ixora chinensis*	Rubiaceae	Ornamental	Kaisoon et al., 2011
*Ixora coccinea*	Rubiaceae	Ornamental	Wongwattanasathien et al., 2010
*Lantana camara*	Verbenaceae	Ornamental	Li et al., 2014
*Lavandula angustifolia*	Lamiaceae	Medicinal and Ornamental	Zeng et al., 2014
*Leucaena leucocephala*	Fabaceae	Ornamental	Kaisoon et al., 2011
*Lilium bulbiferum*	Liliaceae	Ornamental	Zeng et al., 2014
*Limonium sinuatum*	Plumbaginaceae	Ornamental	Li et al., 2014
*Lonicera japonica*	Verbenaceae	Ornamental	Zeng et al., 2014
*Matricaria chamomilla*	Asteraceae	Medicinal	Singh et al., 2011
*Malva sylvestris*	Malvaceae	Medicinal	Loizzo et al., 2016
*Millingtonia hortensis*	Bignoniaceae	Ornamental	Wongwattanasathien et al., 2010
*Mimulus* x *hybridus*	Phrymaceae	Ornamental	Grzeszczuk et al., 2018
*Nelumbo nucifera*	Nelumbonaceae	Ornamental	Kaisoon et al., 2011
*Malva sylvestris*	Malvaceae	Medicinal	Barros et al., 2010
*Monarda*	Lamiaceae	Ornamental	Grzeszczuk et al., 2018
*Oxalis corymbosa*	Oxalidaceae	Ornamental	Li et al., 2014
*Pelargonium peltatum*	Geraniaceae	Ornamental	Benvenuti et al., 2016
*Petunia* x *hybrida*	Solanaceae	Ornamental	Benvenuti et al., 2016
*Plumeria obtusa*	Apocynaceae	Ornamental	Kaisoon et al., 2011
*Primula vulgaris*	Primulaceae	Wild and Ornamental	Kalemba-Drożdż and Cierniak, 2019
*Prunella vulgaris*	Lamiaceae	Wild	Zeng et al., 2014
*Punica granatum*	Punicaceae	Ornamental and Food as Fruit	Wongwattanasathien et al., 2010
*Rhinacanthus nasutus*	Acanthaceae	Ornamental	Wongwattanasathien et al., 2010
*Rhododendron simsii*	Ericaceae	Ornamental	Li et al., 2014
*Robinia pseudoacacia*	Fabaceae	Ornamental	Mlcek and Rop, 2011
*Rosa odorata*	Rosaceae	Ornamental crop	Rop et al., 2012
*Salvia pratensis*	Lamiaceae	Wild and Ornamental	Kucekova et al., 2013
*Salvia splendens*	Lamiaceae	Ornamental	Li et al., 2014
*Sambucus nigra*	Caprifoliaceae	Wild and Ornamental	Kucekova et al., 2013
*Strelitzia reginae*	Strelitziaceae	Ornamental	Li et al., 2014
*Syringa vulgaris*	Oleaceae	Ornamental	Mlcek and Rop, 2011
*Syzygium malaccense*	Myrtaceae	Ornamental	Wongwattanasathien et al., 2010
*Tagetes erecta*	Asteraceae	Ornamental	Benvenuti et al., 2016
*Tagetes patula*	Asteraceae	Ornamental	Kalemba-Drożdż and Cierniak, 2019
*Tamarix gallica*	Tamaricaceae	Ornamental	Ksouri et al., 2009
*Taraxacum officinale*	Asteraceae	Wild and Medicinal	Kucekova et al., 2013
*Telosma minor*	Apocynaceae	Ornamental	Kaisoon et al., 2011
*Tragopogon pratensis*	Asteraceae	Wild	Kucekova et al., 2013
*Trifolium pratense*	Fabaceae	Wild and Forage	Kalemba-Drożdż and Cierniak, 2019
*Trifolium repens*	Fabaceae	Wild and Forage	Kucekova et al., 2013
*Tropaeolum majus*	Tropaeolaceae	Ornamental	Benvenuti et al., 2016
*Tulipa* spp.	Liliaceae	Ornamental	Mlcek and Rop, 2011
*Viola* x *wittrockiana*	Violaceae	Ornamental	Benvenuti et al., 2016
*Viola arvensis*	Violaceae	Wild and Ornamental	Kucekova et al., 2013
*Viola cornuta*	Violaceae	Ornamental	Kalemba-Drożdż and Cierniak, 2019
*Viola tricolor*	Violaceae	Wild	Koike et al., 2015
*Zingiber* spp.	Zingiberaceae	Horticultural	Rachkeeree et al., 2018

**FIGURE 1:**
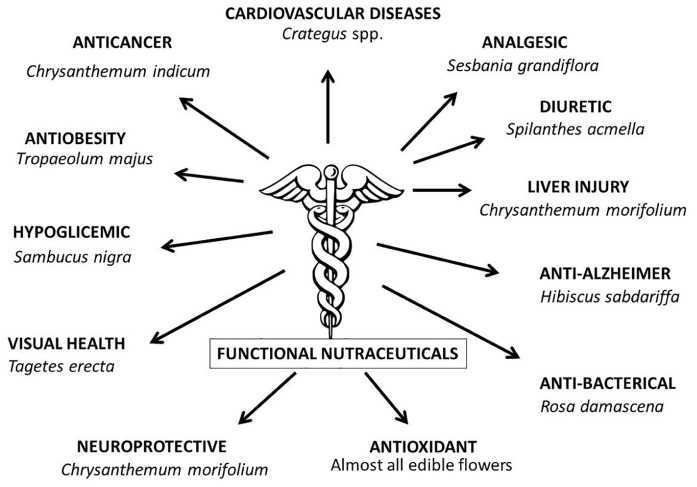
Functional nutraceuticals scientifically proven on human health.

In parallel with this widespread nutraceutical property, phytochemical and pharmaceutical experiments have shown that the flowers have a wide range of medicinal properties against specific pathologies (Figure 1). For example, it has been scientifically proven (Ukiya et al., 2002) that chrysanthemum flowers (*Chrysanthemum morifolium*) and other ornamental species (Pires et al., 2018) have anticancer properties. Similar nutraceutical activities have been demonstrated in a wide range of species that are “functional” to particular pathologies such as hypoglycemic (Loizzo et al., 2016), antimicrobial (Ksouri et al., 2009; Fernandes et al., 2017), anti-Alzheimer (Rezende et al., 2019), the prevention of liver injury (Sugawara and Igarashi, 2009), analgesic (Loganayaki et al., 2012), anti-obesity (Kim et al., 2017), visual health (Nwachukwu et al., 2016), neuroprotective (Ma and Wako, 2017), anti-bacterial (Pires et al., 2018), anti-obesity (Kim et al., 2017), and diuretic properties (Ratnasooriya et al., 2004). They can also help combat cardiovascular diseases (Koch and Malek, 2011). Flowers thus offer a wide range of phytochemical benefits to human health. Integrating them into a daily or periodic diet can help prevent generalized (i.e., antioxidant activity) and/or specific pathologies (Gostin and Waisundara, 2019).

## Flower Chemicals Responsible for the Nutraceutical Activity

The considerable nutraceutical activity in terms of antioxidant power of flowers derives from their richness in generic phenolic compounds (Xiong et al., 2014). This biological activity (Ryu et al., 1994) is proportional to the content in carotenoids, flavonoids (especially anthocyanins), simple phenolic acids and also to vitamins and essential oils (Mlcek and Rop, 2011). From this wide range of phytochemicals, flavonoids, consisting of flavonols, flavones, and anthocyanins show a strong biological activity (Lu et al., 2016). These chemicals play a crucial role in mitigating the oxidative stress induced by various pathologies (Ishige et al., 2001). In relation to this, anthocyanins are particularly important since highly pigmented flowers have a high antioxidant activity (Khoo et al., 2017) compared to cultivars of the same species characterized by less pigmented flowers.

It is no coincidence that the flowers are particularly rich in these phytochemicals since the name anthocyanins derives from the ancient Greek “anthos” (=flower) and “kyáneos” (=blue). These water-soluble substances confer the characteristic red, purple or blue colors according to the pH and their structural features (Fossen and Andersen, 2003). Foods rich in these phytochemicals, especially flowers and fruits, are substances now considered as a real “pharmaceutical ingredients” with great benefits for human health (Khoo et al., 2017). Other pigments of crucial nutraceutical importance belong to the category of carotenoids which bestow yellow and orange colors. Flowers with these colors, such as the different species belonging to the botanical genus *Tagetes*, are particularly rich in violaxanthins, luteins, zeaxanthins, α-carotenes, and β-carotenes (Park et al., 2017).

Among carotenes, the lutein concentration (Niizu and Rodriguez-Amaya, 2005) has active therapeutic benefits, particularly for the human eye. Very lutein-rich flowers are typical of species such as *Tagetes erecta* (Šivel et al., 2014) and *Tropaeolum majus* (Niizu and Rodriguez-Amaya, 2005).

A further category of nutraceutical substances consisting of simple phenolic acids should also be considered. In fact phenolic acids and flavonoids are the most common phenolic compounds with prevalent nutraceutical activity. These substances are very widespread in flowers, especially in the botanical genus *Rosa* (Zheng et al., 2018), and contribute energetically to antioxidant power. For example, in ornamental species such as *Tropaeolum majus*, *Tagetes erecta*, and *Spilanthes oleracea* the phenolic content is closely related to the overall antioxidant activity found in the various species (Navarro-González et al., 2015). This biological activity is also due to the content of vitamins A, C, and E in the flower tissues (Mlcek and Rop, 2011), such as in rose (*Rosa hybrida*) petal extracts (Hou et al., 2014). These flower vitamins could limit or prevent nutritional deficiencies due to a prolonged standardized diet (Rop et al., 2012). In particular, vitamin E was found to consist mainly of four tocopherols (α-, β-, γ-, and δ-tocopherol) and two tocotrienols (β- and γ-tocotrienol) in *Borago officinalis*, *Camellia japonica*, *Centaurea cyanus*, and *Viola* x *wittrockiana* (Fernandes et al., 2020).

A further phytochemical category that characterizes flowers is that of essential oils, which are a very complex natural mixture with a high content of terpenes (Bakkali et al., 2008). Essential oils are not exclusive to aromatic species, as in the case of basil (Chalchat and Özcan, 2008), but are also widespread in many other ornamental species, such as *Chrysanthemum indicum* (Lee et al., 2015), thus generating interest both for food uses and pharmaceutical applications (Voon et al., 2012). Besides being primarily responsible for imparting aroma, essential oils also have a strong antimicrobial activity.

Finally, the mineral content of flowers is significant (Fernandes et al., 2017) both in terms of macronutrients (phosphorus, potassium, calcium, and magnesium) and micronutrients (iron, manganese, copper, and zinc). For example, flowers belonging to the botanic genus *Chrysanthemum*, *Dianthus* or *Viola* are particularly rich in these substances, especially in terms of potassium (Rop et al., 2012). Some species belonging to the botanical genus *Monarda* are also very rich in calcium and magnesium (Grzeszczuk et al., 2018). However, zinc, which is particularly found in *Tagetes patula* flowers (Rop et al., 2012), content is of nutraceutical importance, since zinc is heavily involved in modulating the immune function (Haase and Rink, 2014).

It should be emphasized that these nutraceutical phytochemicals can be useful not only as food but also as drugs following recent processing and extraction of bioactive compounds by novel technologies (Zhao et al., 2019).

## Ecological Role of Flower Phytochemicals

**FIGURE 2:**
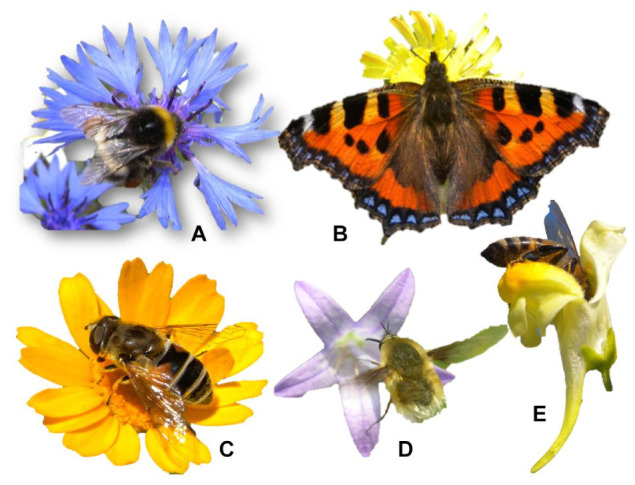
Chromatic flower attraction for pollinators [**(A)** bumblebee on *Centaurea cyanus*; **(B)** butterfly on *Taraxacum officinale*; **(C)** hoverfly on *Glebionis segetum*; **(D)** bee fly on *Campanula rapunculus*; **(E)** domestic bee on *Linaria vulgaris*] as examples of mutualistic flora-fauna co-evolution.

Almost all ornamental flowers have evolved chromatically showy corollas as a strategy to attract pollinators [mainly bees, solitary bees, bumblebees, hoverfly (Diptera Syrphidae), bee fly (Diptera Bombyliidae), and butterfly], since they are responsible for the gene flow within each species (Figure 2). Indeed this showiness is a sort of “publicity” to facilitate their identification. The “eye catching” corollas stand out in the green of the vegetation behind. Plant biomass is dominated by the color green due to the chlorophyll pigments, and the other pigments (flavonoids and carotenoids) that develop corollas, facilitate flower recognition. This is why large and highly pigmented flower species, which have evolved as an entomogamy strategy (Vanbergen and Initiative, 2013), is the portion of plant biodiversity that has aroused the most interest as ornamental use. Very often pigments such as carotenoids and anthocyanins are typically abundant in species that have little or no possibility of self-pollination due to pollen incompatibility within the same plant (Busch and Schoen, 2008), as occurs in *Centaurea cyanus* (Bellanger et al., 2015).

The high pigmentation of the flower corollas derives from a co-evolution mechanism with a mutual reward: (i) advertising for pollinators by increasing the degree of seed set and (ii) evolution of the visual system of pollinators thus facilitating the food detection (pollen and / or nectar). This also happens in intensely colored fruits (blackberries, blueberries, raspberries, etc.) in which the pigmentation derives from a flora-fauna co-evolution that facilitates fruit recognition and attractiveness thus leading to frugivorous dispersal (Shanahan et al., 2001). In this case the reward for frugivores consists of the fruit tissues surrounding the seeds. Flower and fruit pigmentation is thus a real “advertising” strategy for flowers and fruits, which are more easily identified in the green background of the leaf canopy.

From a phytochemical point of view, the wide range of possible colors is generated by the quantity and quality of three categories of pigments: carotenoids (conferring red and yellow colors), anthocyanins (purple and blue), and other flavonoids (white and yellow). The range of flower colors used as a “recognition strategy” is not accidental, since some colors are frequently used while others are decidedly less chosen by the various species. This is because insects have a visual system which, depending on the category of insect considered (bees, Diptera, Lepidoptera, etc.), with an optimal “vision” in certain bands of the light spectrum and overall in the ultraviolet region (Chittka and Raine, 2006).

In addition to the bright chromaticity of the flower corollas, flower scents also play an important role in terms of attractiveness (Pichersky and Gershenzon, 2002). The ecological strategy of emitting highly volatile substances, in order to be visualized at a distance, was subjected to co-evolution between flowers and pollinators (Dötterl and Vereecken, 2010). Does this therefore mean that there is also something in common with pollinators in terms of organoleptic taste perception? It is surprising that this makes flowers attractive to humans too, given that they are the play of colors and the scents showing the same attractiveness perceived by many pollinators, especially bees and bumblebees (Leonard and Masek, 2014).

The scent of flowers derives from a great complexity of chemical substances (Knudsen et al., 2006), which belong to different classes of compounds, such as aliphatics, benzenoids, phenylpropanoids, and terpenes (mono- and sesquiterpenes). The most common “highly volatile” compounds are made up of some monoterpenes such as limonene, (E) -β-ocimene, myrcene, linalool, α- and β-pinene. Benzenoids are also very widespread such as benzaldehyde, methyl 2-hydroxybenzoate (methyl salicylate) as well as benzyl alcohol, 2-phenyl ethanol, and sesquiterpene caryophyllene.

To sum up, insect pollinated flora has evolved a chromatic and olfactory attraction not only for pollinators, but also for humans. Indeed flower shapes, colors and scents generate a high emotional impact (Haviland-Jones et al., 2005), whose psychological aspects have become synergistic with the nutritional ones.

## Nutritional Value of the Different Flower Structures

**FIGURE 3:**
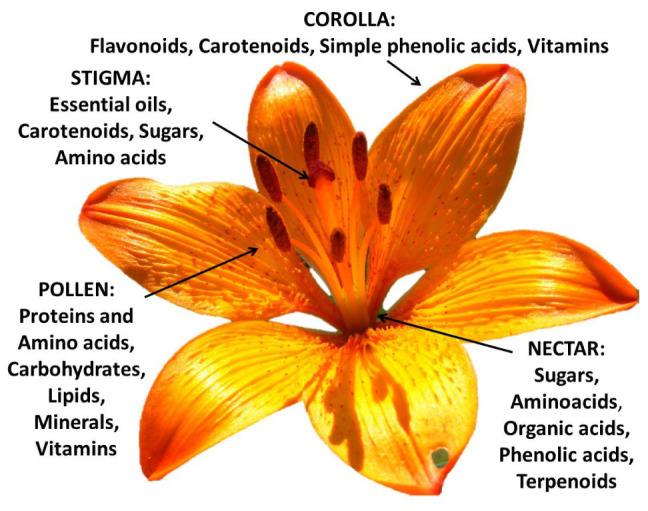
Prevalent phytochemicals of the different components of the edible *Lilium bulbiferum* flower (corolla, stigma, pollen, and nectar).

The heterogeneity of the botanical structures of several species of flowers is closely connected with their respective chemical diversity (Figure 3). This phytochemical complexity makes edible flowers especially interesting. Their nutritional value is provided by the pollen (rich in proteins and amino acids), nectar (rich in sugars), and corolla tissues (rich in pigments, vitamins, and microelements). This mixture of primary (sugars, proteins, etc.) and secondary metabolites (vitamins, pigments, etc.) helps prevent nutritional deficiencies in the human diet (Pires et al., 2019). Below we analyze the phytochemical properties of each flower component.

Although constituting a small part of the flower, pollen is mostly rich in carbohydrates (13–55%), protein and amino acids (10–40%), and to a lesser extent in polyunsaturated lipids (1–13%), fiber and pectins, (0.3–20%), minerals (2–6%) and small amounts of other chemical substances including some important vitamins (2–5%) such as β-carotene, thiamine, riboflavin, niacin, pantothenic acid, ascorbic acid, biotin, folic acid, and tocopherols (Campos et al., 2008). This phyto-complex indicates an important nutraceutical role of its bioactive properties for human health (Kroyer and Hegedus, 2001). Pollen shows antioxidant, anti-inflammatory, anticarcinogenic, antibacterial, antifungicidal, hepatoprotective, anti-atherosclerotic activities and modifies or regulates immune functions (Denisow and Denisow-Pietrzyk, 2016). It should be emphasized that although pollen is capable of generating allergies, this typically occurs in wind-pollinated species characterized by very small pollen capable of aerodispersion (Culley et al., 2002). On the contrary, the insect-pollinated species (as in the case of ornamental species) are characterized by larger pollen that can hardly cause allergy problems. On the other hand the use of pollen as a food rich in nutraceutical properties is well known (Llnskens and Jorde, 1997).

Despite its limited content in the whole flower, the nectar constitutes a balanced mixture of amino acids and sugars consisting of fructose, glucose, and sucrose (Pacini et al., 2003). The nectar also contains secondary metabolites such as organic acids, simple phenolic acids, and terpenoids (Nicolson et al., 2007). Although stigmas are less important and poorly studied as a component of edible flowers, they have been found to contain essential oils in the case of *Cucurbita pepo* (Granero et al., 2005) and carotenoids in the well-known *Crocus sativus* (Melnyk et al., 2010). In addition, sugars and amino acids have been detected in the stigmatic secretions of pomaceous flowers (Pusey et al., 2008).

Lastly, the corolla is the main portion of the flower in terms of biomass. Consequently, the quantity and chemical composition of the phytochemicals present in their tissues provide a nutraceutical functionality to a specific species. This typically highly pigmented flower portion contributes most of the health properties in terms of antioxidant activity thanks to the wealth of anthocyanins, other flavonoids, carotenoids, simple phenolic acids, ascorbic acid (Garzón and Wrolstad, 2009), and other vitamins (Fernandes et al., 2020). This nutraceutical role of the corollas has meant that most species used as edible flowers have also been selected due to their flower size, as in the case of *Nasturtium officinale*, *Petunia* spp., *Tagetes* spp., *Antirrhinum majus*, and *Viola* x *wittrockiana*. However, the success of some of the many species with potentially edible flowers is due to the organoleptic impact perceived by consumers.

## Discovering the Unknown Taste of Flowers

The growing interest in edible flowers is motivated not only by decorative and nutraceutical objectives, but also by the desire for new flavors and new opportunities for gastronomic innovation. Some studies have identified the sensory profile of the flowers (Benvenuti et al., 2016), and these “panel tests” have highlighted not only the level of overall appreciation of some species of flowers but also the individual organoleptic characteristics (sweetness, spiciness, aroma, bitterness, consistency) as well as their similarity with other already known vegetables and/or spices. The flowers that were particularly appreciated were *Tropaeolum majus*, characterized by the spicy taste (similar to that of radish), while *Ageratum houstonianum* aroused a taste that was similar to a carrot. The *Begonia semperflorens* was also of particular interest, which was decidedly sour, similar to lemon juice.

Other promising organoleptic results (Benvenuti et al., 2016) have been shown by *Dianthus* x *barbatus* which tastes similar to cloves, *Calendula officinalis* which is similar to saffron, and *Pelargonium peltatum* similar to grapefruit. Other species, have been perceived as having a completely new taste such as the flowers of *Petunia hybrida* and *Viola* x *wittrockiana*. In some cases the consistency of the flower was not liked, for example *Fuchsia hybrida*, however, a gastronomic analysis of these flowers (comminution, seasoning, mixed with other vegetables, cooking, etc.) might improve their palatability. Overall the flowers that seem to simultaneously satisfy aroma and consistency are *Nasturtium officinale*, *Viola* x *wittrockiana*, *Ageratum houstonianum*, and *Begonia semperflorens*.

Tasting these “new vegetables” has generated a mixture of enthusiasm and curiosity, together with an understandable mistrust in the strange shapes, consistency, flavors and aromas. People’s negative attitudes toward new foods comes under the term “neophobia.” This mistrust of new food could be mitigated by food education in children, which is the age when our taste perception begins to be formed (Mustonen and Tuorila, 2010). The discovery of new flavors, especially in children, could stimulate the appreciation of flowers, thus preventing them from being regarded as too unusual. In fact, an innate instinct to avoid unknown tastes hinders a full success of this “new food” (Pliner and Hobden, 1992) especially in children (Dovey et al., 2008). Flowers need to be combined with other foods in order to make their acceptance easier. The optimal combinations between flower dishes and a specific type of wine or beer could also help to enhance each other’s characteristics.

## Which Flower Species Are Current or Potential “New Foods”?

As to which flowers can be eaten, there are no clear boundaries between edible species, and it depends on the taste of the consumer. The only objective characteristic is their non-toxicity, which will be examined in the next section. To limit the species with edible flowers there are various books (Barash, 1993; Roberts, 2000) and scientific publications (Table 2) which list the flowers with traditional ethnobotanical uses as food (Mulík and Ozuna, 2020) and/or whose phytochemical aspects have been recently discovered.

**TABLE 2:** Poisonous, toxic, and anti-nutritional activity of ornamental species.

Scientific name	Botanic family	Main phytochemicals	Degree of toxicity	References
*Convallaria majalis*	Liliaceae	Glycosides	Toxic	Löffelhardt et al., 1979
*Asphodelus* spp.	Liliaceae	Glycosides	Toxic	Safder et al., 2009
*Ornithogalum* spp.	Liliaceae	Glycosides	Toxic	Botha et al., 2000; Slenter et al., 2019
*Colchicum autumnale*	Liliaceae	Alkaloids	Poisonous	Klintschar et al., 1999
*Conium maculatum*	Apiaceae	Alkaloids	Poisonous	Vetter, 2004
*Laburnum anagyroides*	Fabaceae	Alkaloids	Poisonous	Schep et al., 2009
*Narcissus* spp.	Amaryllidaceae	Alkaloids	Toxic	Bastida et al., 2006
*Digitalis* spp.	Scrophulariaceae	Glycosides	Poisonous	Yang et al., 2012
*Datura* spp.	Solanaceae	Alkaloids	Toxic	Krenzelok, 2010
*Atropa belladonna*	Solanaceae	Alkaloids	Toxic	Kwakye et al., 2018
*Euphorbia pulcherrima*	Euphorbiaceae	Terpenes	Toxic	Smith-Kielland et al., 1996
*Vinca* spp.	Apocynaceae	Alkaloids	Toxic	Gutowski et al., 1995
*Nerium oleander*	Apocynaceae	Glycosides	Poisonous	Barbosa et al., 2008
*Hyoscyamus* spp.	Solanaceae	Alkaloids	Toxic	Shams et al., 2017
*Anemone* spp.	Ranunculaceae	Alkaloids	Toxic	Turner, 1984
*Ranunculus* spp.	Ranunculaceae	Alkaloids	Toxic	Turner, 1984
*Helleborus* spp.	Ranunculaceae	Alkaloids	Toxic	Turner, 1984
*Aconitum napellus*	Ranunculaceae	Alkaloids	Toxic	Turner, 1984
*Yucca filifera*	Liliaceae	Inhibitory enzymes	Anti-nutritional	Sotelo et al., 2007
*Erythrina* spp.	Fabaceae	Inhibitory enzymes	Anti-nutritional	Sotelo et al., 2007
*Cyclamen* spp.	Primulaceae	Glycosides	Toxic	Spoerke et al., 1987
*Paeonia* spp.	Paeoniaceae	Alkaloids	Toxic	Sadati Lamardi et al., 2018
*Rhododendron* spp.	Ericaceae	Terpenoids	Toxic	Popescu and Kopp, 2013
*Mirabilis jalapa*	Nyctaginaceae	Inhibitory enzymes	Anti-nutritional	Kowalska et al., 2007
*Jasminum* spp.	Oleaceae	Glycosides	Toxic	Bhushan et al., 2014
*Callistemon citrinus*	Myrtaceae	Terpenes	Toxic	Bhushan et al., 2014
*Hydrangea macrophylla*	Hydrangeaceae	Glycosides	Toxic	Ito et al., 2009
*Wisteria sinensis*	Fabaceae	Glycosides	Toxic	Mohamed et al., 2011
*Cytisus scoparius*	Fabaceae	Alkaloids	Toxic	Wink et al., 1982

Most species used as edible flowers are ornamental species. This derives both from their wide availability, as widely cultivated species, and due to the fact that their ornamentality is closely linked to their large corollas and/or the high number of flowers or inflorescences per plant. Species with large-sized flowers are, for example, *Hibiscus rosa-sinensis*, *Lilium bulbiferum*, *Petunia* x *hybrida*, *Tagetes erecta*, *Tropaeolum majus*, and *Viola* x *wittrockiana*.

Other species have smaller but extremely numerous flowers such as *Oxalis corymbosa*, *Impatiens walleriana*, *Dianthus* x *barbatus*, *Limonium sinuatum*, *Viola cornuta*, *Ageratum houstonianum*, and *Agastache foeniculum*.

Some species with edible flowers, although sometimes also used as ornamentals, are typical medicinal crops such as *Matricaria chamomilla*, *Calendula officinalis*, *Taraxacum officinalis*, and *Lavandula angustifolia*. In these cases the flowers are gathered in inflorescences and are small but very aromatic, and can be used in chromatically and aromatically innovative gastronomic creations.

In other cases, the edible flowers belong to common horticultural species which have been traditionally (*Cucurbita pepo*), or recently (*Cichorium inthybus*), used as food. There are also species with edible flowers that are cultivated as forage such as *Trifolium repens and Trifolium pratense*.

Finally, there is a vast biodiversity of wild species which are also sometimes cultivated similarly to what occurs for wild herbs (Schulp et al., 2014). These are species found in natural (or partially anthropized) ecosystems such as *Bellis perennis*, *Borago officinalis*, *Campanula rapunculus*, *Allium schoenoprasum, Anthirrhinum majus*, and *Prunella vulgaris*. Sometimes species with edible flowers even include weeds of traditional agro-ecosystems such as *Centaurea cyanus* and *Viola tricolor*. These insect-pollinated weeds are often rare and used as an “indicator” of the health and biological sustainability of agro-ecosystems (Rollin et al., 2016), and consequently their food use could facilitate their germplasm conservation.

## Which Species Should Be Avoided?

Some plant species have evolved toxicity and/or poisonousness in order to defend themselves from both parasitic and/or pathogenic organisms and, above all, from herbivorous fauna. Consequently, great care must be taken not to confuse the beauty of the flowers with their usability as food since some may be toxic or poisonous for both humans and animals (Tamilselvan et al., 2014).

The phytochemicals responsible for toxicity belong above all to the chemical categories of alkaloids, saponins, terpenes and glycosides (Mithöfer and Boland, 2012). These species mainly belong to the botanical families of liliaceae, amaryllidaceae, apiaceae, fabaceae, scrophulariaceae, solanaceae, euphorbiaceae, apocynaceae, and ranunculaceae (Table 2). While in some botanical families, almost all species have frequently high toxicity and/or poisonousness (such as ranunculaceae, apocynaceae, euphorbiaceae, and ranunculaceae), the toxic species of other botanical families such as liliaceae, fabaceae, scrophulariaceae and solanaceae are rare, while many others, belonging to these botanical *taxa*, are widely used as food or as medicine, including both the flowers and other parts of the plant.

The ranunculaceae family is one example of a botanical family rich in ornamental species but which are almost always toxic. These include the various species belonging to the botanical genus *Anemone* (*Anemone coronaria*, *Anemone hortensis*, *Anemone nemorosa*, etc.) the genera *Ranunculus*, *Aquilegia*, *Helleborus* and above all the poisonous species belonging to the genus *Aconitum*.

Other botanical families have species whose flowers are edible, but other species that are toxic. For example the family of liliaceae includes species with edible flowers (for example *Lilium bulbiferum*, *Allium schoenoprasum*, *Tulipa* spp.). While in other cases the ingestion of the flowers can cause toxicity (for example *Convallaria majalis*, *Asphodelus* spp., *Ornithogalum* spp., and *Colchicum autumnale*).

Another real risk for humans is toxic nectar (Adler, 2000), since in some cases pyrrolizidine alkaloids have been detected in honey (Edgar et al., 2002; Dübecke et al., 2011). This problem appears to be linked above all to species belonging to the botanical family of Boraginaceae such as in *Heliotropium amplexicaule* (Carpinelli de Jesus et al., 2019) and *Echium vulgare* (Lucchetti et al., 2016). Such hepatotoxic pyrrolizidine alkaloids have also been detected in pollen, although to date this problem has only been detected in a few species (Kast et al., 2018) belonging to the botanical genus *Echium* and in species belonging to other families such as Asteraceae (*Senecio* spp. and *Eupatorium cannabinum*).

Additional interest in edible flowers is due not so much to the toxic phytochemicals but to anti-nutritional phytochemicals capable of altering the normal metabolism (Sotelo et al., 2007). For example, a study carried out in Mexico on various “endangered” species, showed that although the splendid flowers of the *Yucca filifera*, which are sometimes used as a food in local dishes, contain undesirable saponins with hemolytic activity. *Agave salmiana*, on the other hand, shows hemagglutinating activity (Barriada-Bernal et al., 2014). In addition, some species of fabaceae belonging to the botanical genus *Erythrina* (*Erythrina americana* and *Erythrina caribaea*) contain trypsin inhibitor enzymes.

Further studies are thus needed to reliably identify those species that are truly edible. However, some species that are “to be avoided” may also have a purpose as, as according to the Swiss doctor, Paracelsus (1493-1541): “The dose makes the poison.” There are in fact many uses of toxic flowers as medicines, which could constitute a further innovation chain in the agronomic and pharmaceutical sector.

## Which Cropping Systems Are Suitable for Edible Flowers?

Producing edible flowers clearly requires organic cultivation systems (Kelley and Biernbaum, 2000) since current and potential consumers are particularly attracted to “novel foods” that are both nutraceutical and completely pesticide free. In fact some studies (Chen, 2009) have shown that a healthy lifestyle mediates health consciousness and attitude toward organic foods. This is why some “nutrient management” techniques focused on organically grown edible flowers (Kelley and Biernbaum, 2000) have been tested in the same way as conventional vegetables (Van Bruggen et al., 2016). However, similarly to common leaf vegetables for fresh consumption, flowers must ensure food safety also from a biological point of view, which could be jeopardized by the use of organic fertilizers that are often rich in unwanted microorganisms (Sharma and Reynnells, 2018). This is particularly important for all ready-to-eat vegetables (Sagoo et al., 2001).

The methods of distribution of organic fertilizers must not come into contact with the epigean part of the plant in any way, thus preventing them from being contaminated in terms of the microbial load.

Unfortunately there have not been sufficient studies on verifying the food safety in microbiological terms of edible flowers grown with organic cropping systems. This is of key importance since one study has verified that flowers grown without the aid of any pesticide are safe from a chemical point of view, but not always a biological one. In fact, unwanted microorganisms such as *Enterobacter hormaechei, Acinetobacter calcoaceticus, Enterobacter ludwigii, Enterobacter asburiae, Enterobacter cowanii, Pseudomonas aeruginosa, Salmonella enterica*, and *Bacillus amyloliquefaciens* (Wetzel et al., 2010) have sometimes been found on “organically grown” flowers. Consequently, prevention from the microbiological contamination of edible flowers is a major problem that merits further studies to ensure greater food safety.

In addition to microbiological contamination, pesticide residues such as sulphite, dimethoate and N, N-diethyl-meta-toluamide (Matyjaszczyk and Śmiechowska, 2019) are extremely undesirable chemicals that are widely used in conventional cropping systems of ornamental crops not intended for human consumption. However, adopting the wide range of crop defense strategies for edible flower production using organic methods is a positive step (Dayan et al., 2009). A further agronomic strategy is to identify species and cultivars that are less susceptible to pathologies such as, the botanic genus *Rosa* (Friedman et al., 2010).

Lastly, the vast biodiversity of species with edible flowers has different biological characteristics both in terms of thermal requirements, photoperiod, biological cycle (annual and perennial), and time and duration of flowering. This complexity involves a diversified agronomic management especially according to the reachable size of the various crops since they are not exclusively herbaceous but also include shrubs and trees. For example ornamental crops such as *Rosa* spp., *Camellia japonica*, *Hibiscus rosa-sinensis*, *Sambucus nigra*, and *Bougainvillea hybrida* are shrubs, while are *Robinia pseudo-acacia* and *Cassia siamea* are ornamental trees with edible flowers. These species are thus almost exclusively cultivable in the open air and therefore their flowering calendars are very rigid and not suitable for ensuring the availability of edible flowers throughout the year.

Unfortunately, the seasonality of flower production is not very compatible with their commercial success due to their unavailability for long periods. Edible flowers also have very sensitive organoleptic characteristics and are not suitable for storage or as frozen or dried products. However, most of the species with edible flowers are herbaceous and thus are suitable for cultivation both in the open air and in the greenhouse, thus ensuring much longer flowering calendars. Basically, species mainly belonging to this last category of herbaceous species are the main candidates to respond to market needs, since they are available throughout the year. This amplitude and flexibility of the blooms are crucial for use as ingredients in various dishes for long periods of the year both for “home cooking” and in “gourmet cuisine.”

A parallel activity for edible flowers is to satisfy the needs of urban agriculture (Eigenbrod and Gruda, 2015). Urban cropping systems for edible flowers are capable of providing both: (i) the maximum degree of freshness (ready-to-eat locally produced at “zero-kilometers”) and (ii) an increase in urban biodiversity especially in terms of pollinators. Edible flowers represent the ideotype of “new vegetables” for “urban edible landscapes” (Specht et al., 2014) with a dual role of being both ornamental and a source of nutritious food.

## What Do Consumers Prefer and What Kind of Packaging Should Be Used?

The standard packaging of edible flowers usually involves transparent polyethylene trays in order to enhance their chromatic impact. Depending on the species, the shelf life, is around 7–10 days at around 2–5°C (Kelley et al., 2003). This low temperature keeps the antioxidant activity almost unchanged as demonstrated in *Tropaeolum majus* (Friedman et al., 2005). The organoleptic alteration that occurs during very long storage includes petal abscission and discoloration, flower wilt, dehydration, and tissue browning. However, the shelf life can be extended by adding methylcyclopropene (1 – MCP) which delays senescence since is a non-toxic antagonist of ethylene, which binds to the ethylene receptors, thus interfering with ethylene mediated changes (Kou et al., 2012).

Post-harvest technologies such as high hydrostatic pressure (HHP) or irradiation (Fernandes et al., 2019) will become increasingly important in order to extend the marketing of edible flowers in terms of time and space. However, the commercial success of edible flowers derives not only from further studies on their shelf life, but also from marketing strategies that attract consumers to this surprising and unusual “new food.”

Curiosity and aroma seem to be the parameters that most influence consumer attitudes toward the consumption of edible flowers as being “ready to eat” (Chen and Wei, 2017). Similarly, the combination of colors in the trays displayed on supermarket shelves also plays a crucial role in terms of attractiveness (Kelley et al., 2001). In particular, the combination of blue, yellow, and orange seems successful in influencing consumer choices. Transparent packaging plays a crucial role in highlighting the beauty of the heterogeneity of flower shapes and colors (Chen and Wei, 2017). Immediately after the visual impact of the package, the price is able to influence the consumer’s choices. Finally, the size of the package plays a marginal role in influencing the behavior of potential consumers (Kelley et al., 2001). A further aspect of growing interest is to provide recipes that enhance the attraction of the flowers in three ways: (i) chromatic attractiveness, (ii) tastiness, and (iii) benefit for human health. Of course these may vary according to the cultural differences of the consumers (Rodrigues et al., 2017). The most appropriate criterion is to use well-known gastronomic creations for a specific “local” gastronomic culture (soups, pasta, bread, meat, fish, sweets, salads, ice cream, yogurt, herbal teas, etc.) in order to make more new food more familiar.

## Conclusion

The biodiversity of ornamental and wild species with edible flowers is one of the most promising resources for gastronomic innovation aimed at offering new tastes and new combinations of flowers with other foods (including vegetables and meat, fish and types of beer and wines). Their nutraceutical potential benefits human health also in terms of a psychological impact by combining the beauty of flowers with their tastiness. Future studies will increasingly underline the nutraceutical potential of the various species of flowers that can prevent certain pathologies and thus be part of “personalized diets” aimed at specific health problems.

In summary, this review has highlighted some key aspects that can encourage a further affirmation of edible flowers trying to answer the following questions: which species are currently used (especially ornamental species), which have the characteristics of a future affirmation (many wild species with potential domestication), which ones are avoided (some toxic, poisonous species and / or with anti-nutritional substances), why they are rich in pigments (chromatic strategies to attract pollinators), why they are human health friendly (chemical nature and richness in nutraceutical pigments) and what agronomic perspectives can have (dedicated crops and urban “food” floriculture). Such answers will be able to help, in the near future, the agronomic challenge aimed at making possible the real availability in space and time of these emerging foods. Indeed, although both edible flowers and wild herbs are much cited by important traditional culinary uses, they are still hardly available today in large commercial distribution. Food safety in terms of the absence of both pesticides and pathogenic microorganisms (Nicolau and Gostin, 2015) will be of crucial importance for a complete affirmation of this still unusual food.

In conclusion, edible flowers – ornamental and wild – have the potential of providing an important “ecosystem service” to satisfy the growing desire for new organoleptic discoveries that are healthy both from a biological and psychological point of view.

## Author Contributions

Both authors contributed to the investigation, data curation, supervision, and writing of this review.

## Conflict of Interest

The authors declare that the research was conducted in the absence of any commercial or financial relationships that could be construed as a potential conflict of interest.
